# Dose‐Dependent Effects of Simulated Clinical Radiotherapy on the Structure and Properties of Root Dentine

**DOI:** 10.1111/aej.70053

**Published:** 2026-01-06

**Authors:** Anna Victória Costa Serique, Lívia Ribeiro, Julia Menezes Savaris, Luíz Carlos de Lima Dias‐Júnior, Eduardo Antunes Bortoluzzi, Mariana Comparotto Minamisako, Paulo Marcelo Rodrigues, Nayara Cardoso Cábia, Ricardo Machado, Bruno Alexandre Pacheco de Castro Henriques, Cleonice da Silveira Teixeira, Lucas da Fonseca Roberti Garcia

**Affiliations:** ^1^ Department of Dentistry—Endodontics Division, Health Sciences Center Federal University of Santa Catarina Florianópolis Brazil; ^2^ Endodontics Division, Department of Diagnosis & Oral Health University of Louisville Louisville Kentucky USA; ^3^ Department of Radiotherapy Oncology Research Center (CEPON) Florianópolis Brazil; ^4^ Department of Restorative Sciences, College of Dentistry University of Oklahoma Oklahoma City Oklahoma USA; ^5^ Department of Mechanical Engineering Federal University of Santa Catarina Florianópolis Brazil

**Keywords:** biomechanical properties, head and neck cancer, intensity‐modulated radiotherapy, Raman spectroscopy, root dentine

## Abstract

Head and neck radiotherapy (HNRT) can expose teeth to moderate radiation doses, yet the effects on root dentine under clinically relevant conditions remain unclear. This in vitro study compared dentine subjected to 0, 30, or 50 Gy IMRT. Palatal roots from 35 maxillary molars were allocated to non‐irradiated, 30 Gy (oropharyngeal simulation), or 50 Gy (maxillary simulation). Flexural strength, Vickers microhardness, and Raman spectroscopy were assessed, while SEM was qualitatively evaluated. Mechanical and chemical properties did not differ among groups (*p* > 0.05). SEM, however, revealed dose‐dependent microstructural changes: fissures and partial tubule obliteration at 30 Gy, and more frequent cracks and complete obliteration at 50 Gy. Under these in vitro simulated conditions, radiotherapy doses preserved bulk properties despite structural alterations. These findings should be interpreted within the limitations of the model, as interactions among experimental factors may influence how microstructural changes affect dentine mechanics.

## 1 Introduction

Neoplasms of the oral cavity and oropharynx rank among the most common head and neck cancers (HNC) [1], and their incidence is projected to increase by > 40% between 2020 and 2040 [2]. Early diagnosis and appropriate treatment selection are essential for improving prognosis [3]. Radiotherapy is widely used in HNC management, alone or combined with surgery and chemotherapy [4, 5], producing direct DNA damage [6, 7] and indirect oxidative effects [8]. Standard protocols typically deliver 2 Gy/day, five days per week, for five to seven weeks [9, 10]. Despite advances such as intensity‐modulated radiotherapy (IMRT) [11], which improves targeting precision, surrounding orofacial structures—including bone, mucosa, teeth, and salivary glands—remain vulnerable to radiation exposure [8, 10].

Dentine, a mineralized tissue rich in carbonates and magnesium [10] and composed predominantly of collagen in its organic matrix [4], depends on mineral content for hardness and on collagen for fracture resistance [12], both crucial for maintaining structural integrity [13]. Radiation‐induced caries can cause rapid coronal destruction and pulp changes [14], and because dental extractions increase the risk of osteoradionecrosis [15], preserving tooth structure through endodontic and restorative procedures becomes critical [10, 16]. Importantly, the oral environment of patients undergoing HNC treatment is profoundly altered by mucositis, hyposalivation, dysbiosis, reduced buffering capacity, and changes in diet and oral hygiene [8, 10, 14]. These factors act synergistically with radiation‐induced tissue changes, creating a complex clinical scenario that cannot be reproduced in vitro and that amplifies susceptibility to structural compromise and restorative failures.

Ionising radiation may alter the chemical and mechanical properties of dentine [4, 8, 14, 17], with radiolysis promoting dehydration [10], collagen denaturation [4], and structural weakening that predisposes to fractures [17]. Microstructural changes such as tubule obliteration and odontoblast degeneration can further reduce bonding performance of adhesives and endodontic sealers [8]. High‐dose in vitro studies have demonstrated significant damage to teeth, such as chemical alterations [5], and disruption of mineral‐organic interactions [4] at 72 Gy. Reduced microhardness at 55–70 Gy was also observed by Novais et al. [12] and Velo et al. [10], supporting radiation‐induced weakening of dentine.

Although these findings are consistent, most in vitro studies [4, 8, 12, 14, 17] simulate full therapeutic doses (55–72 Gy), representing the total dose delivered to tumours rather than the dose actually received by adjacent teeth. IMRT beam modulation, tumour site, stage, and laterality influence true dental exposure [15, 18, 19], and radiotherapy planning frequently shows that teeth receive substantially lower doses than those prescribed for tumour control [18, 19]. As a result, laboratory models may overestimate radiation effects on dentine, limiting clinical extrapolation [18, 19]. Understanding dentine alterations according to clinically relevant dosimetry is therefore essential to clarify true dose–response relationships [18]. Simulating real conditions is important for bridging laboratory and clinical findings and strengthening endodontic and restorative protocols for irradiated patients [10, 16]. Therefore, this in vitro study assessed the effects of radiation doses typically delivered to upper molars during maxillary and oropharyngeal cancer treatment on root dentine morphology, chemical composition, and mechanical properties. The null hypotheses were that ionising radiation would not alter: (I) morphology, (II) chemical composition, or (III) mechanical properties of root dentine.

## 2 Materials and Methods

### 2.1 Ethical Concerns, Sample Size Calculation, and Specimens Selection

**FIGURE 1:**
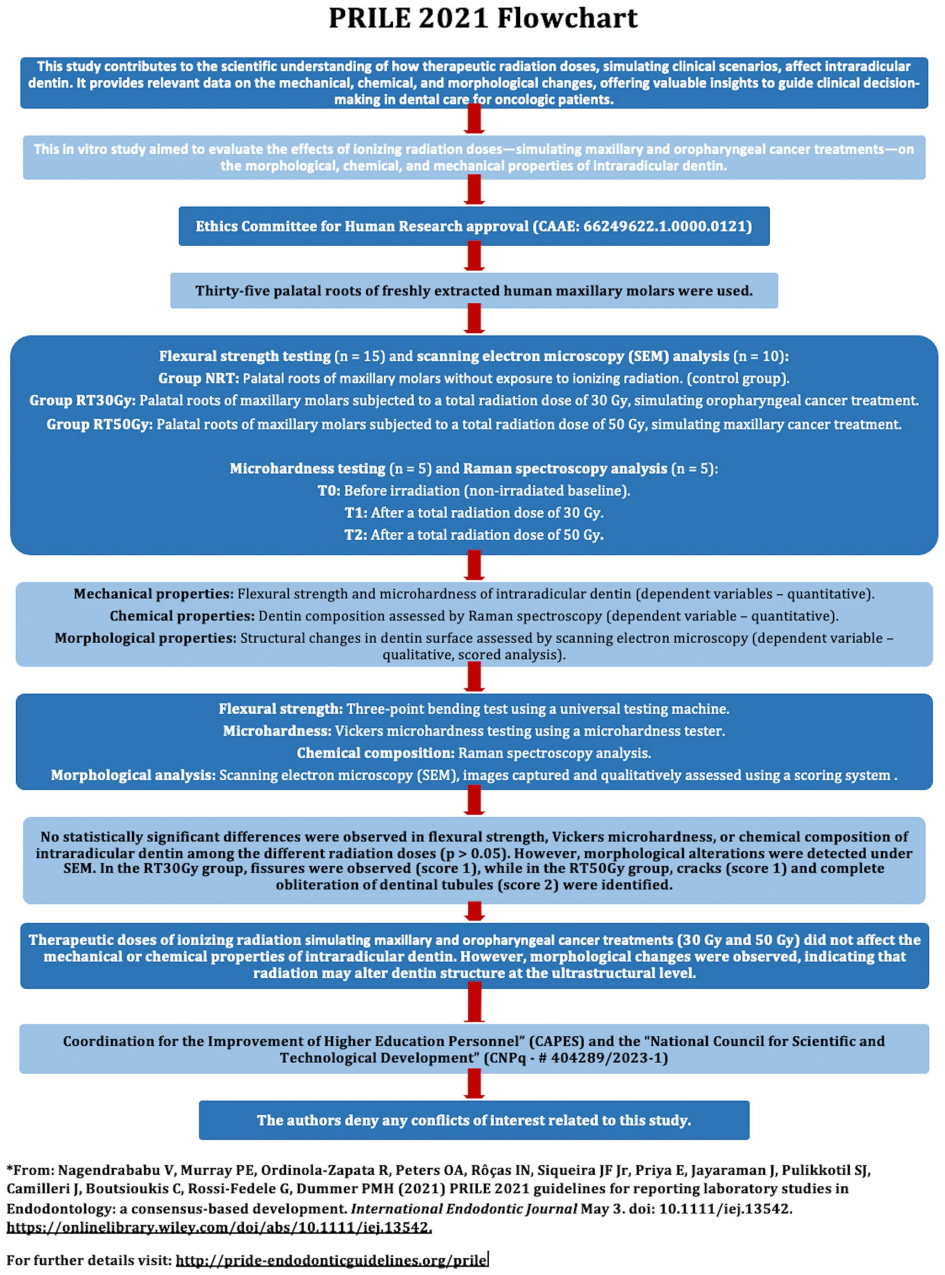
PRILE 2021 reporting framework for in vitro endodontic studies.

The manuscript of this laboratory study has been written according to Preferred Reporting Items for Laboratory studies in Endodontology (PRILE) 2021 guidelines [20] (Figure 1).

Ethical approval was granted by the institutional Ethics Committee (CAAE: 66249622.1.0000.0121). Sample size calculation was performed using G*Power software (version 3.1.9.6; http://www.psycho.uni‐duesseldorf.de/abteilungen/aap/gpower3/) to determine the minimum number of repetitions required for the mechanical tests (*n* = 10)—including three‐point flexural strength and Vickers microhardness—as well as for the morphological (*n* = 5) and chemical (*n* = 10) analyses of root dentine. A 95% confidence level was adopted. The following parameters were considered: α error = 0.05 (significance level = 5%), test power (1‐β) = 0.80, and an effect size = 1 [21, 22]. Thirty‐five human maxillary molars with intact palatal roots were selected. Teeth with caries, cracks, resorption, or previous endodontic treatment were excluded. Specimens were cleaned, disinfected in 0.1% thymol, and stored in distilled water at 37°C until use.

### 2.2 Specimens Preparation and Radiation Therapy Protocol

**FIGURE 2:**
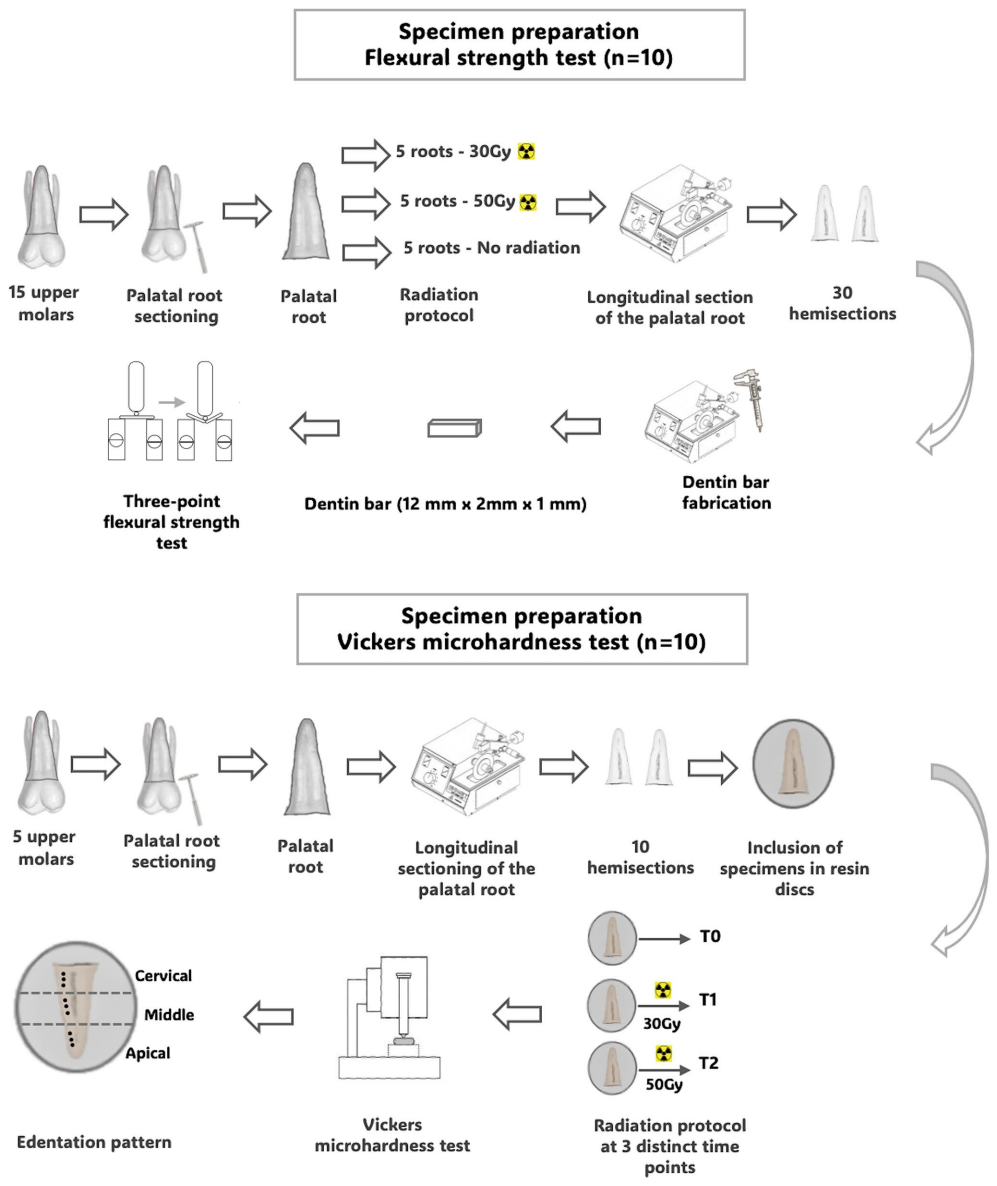
Flowchart of the mechanical tests.

**FIGURE 3:**
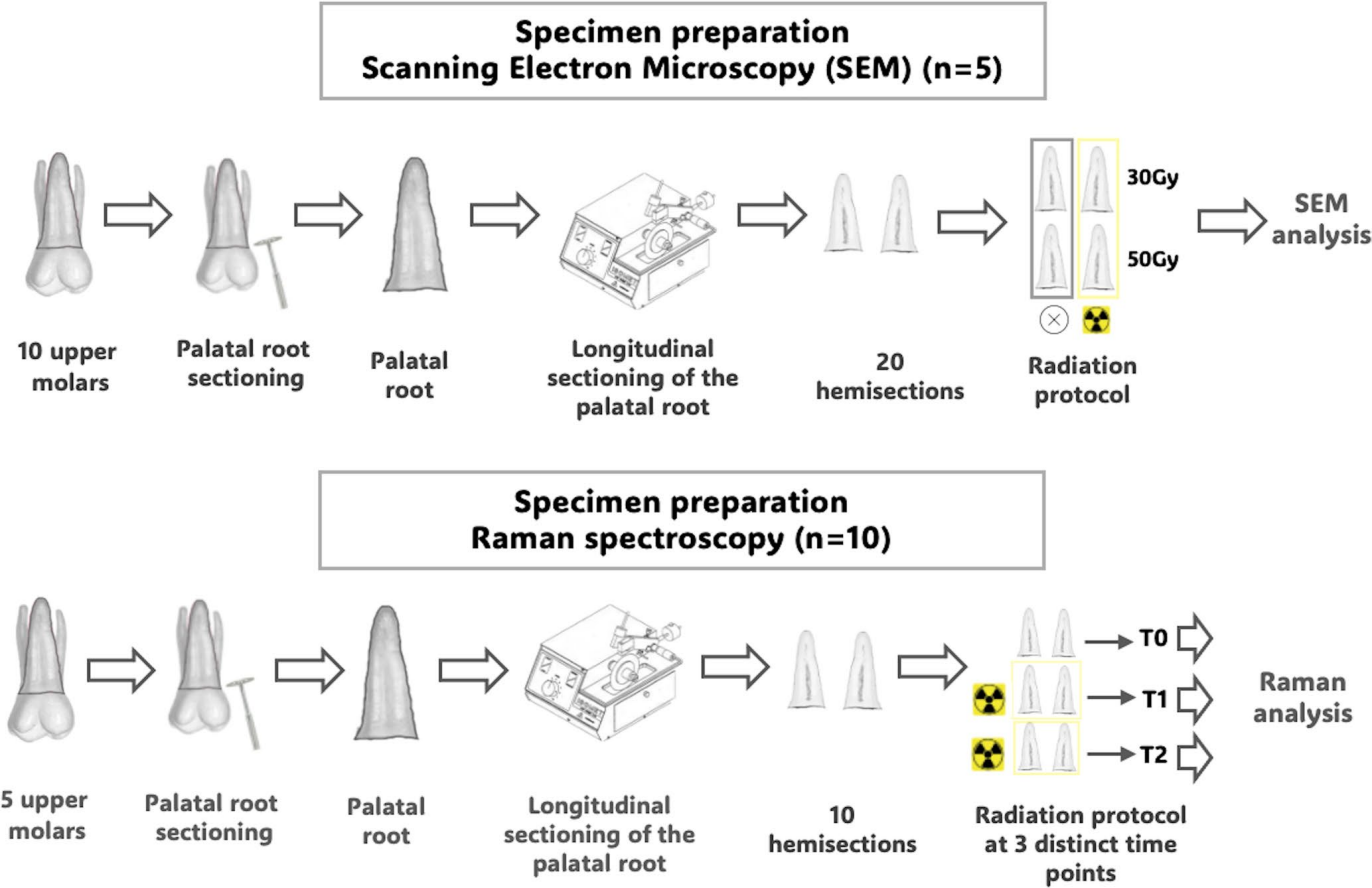
Flowchart of the morphological and chemical analyses.

The crowns of the teeth were sectioned near the cementoenamel junction, followed by the removal of the palatal roots using a double‐sided diamond disc No. 7020 (KG Sorensen, Cotia, São Paulo, SP, Brazil), mounted on low‐speed rotation, under abundant water cooling. The palatal roots were then randomly allocated to the experimental groups (www.random.org). The distribution flowchart for the roots in each experiment is presented in Figures 2 and 3.

Radiation therapy of the specimens was performed using a linear accelerator (Clinac 2100C; Varian Medical Systems Inc., Palo Alto, CA, USA) via IMRT, with dynamic multileaf collimators (DMLC). The roots were placed in a plastic holder containing distilled and deionised water, which was aligned equidistant from the radiation source to ensure standardised distribution of the daily radiation doses (400 UM/min) [23]. The total radiation delivered to the upper molars simulating oropharyngeal cancer treatment (30 Gy) and maxillary cancer treatment (50 Gy) [18] was fractionated into daily doses of 2 Gy, administered five days a week. At the end of each daily irradiation cycle, the distilled and deionised water was replaced with artificial saliva, and the roots were maintained at 37°C to simulate the oral environment. At the beginning of each new irradiation cycle, the artificial saliva was replaced with distilled and deionised water. The entire radiotherapy procedure was carried out at the Department of Radiotherapy of the Oncology Research Center (CEPON, Florianópolis, SC, Brazil), under the supervision of a physicist and a radiation oncologist.

### 2.3 Mechanical Tests

#### 2.3.1 Flexural Strength

For the flexural strength test, fifteen roots were randomly selected and allocated into three groups (*n* = 5): (NRT) negative control (non‐irradiated); (RT30Gy) total radiation dose of 30 Gy delivered to the upper molars, simulating radiotherapy for oropharyngeal cancer; and (RT50Gy) total radiation dose of 50 Gy delivered to the upper molars, simulating radiotherapy for maxillary cancer [18]. The roots were longitudinally sectioned using a high‐precision metallographic cutter (Isomet 1000; Buehler, Lake Forest, IL, USA), producing two hemisections per root, resulting in a total of 10 specimens per group. The hemisections were affixed to an acrylic resin plate with high‐fusion green wax (DFL, Jacarepaguá, Rio de Janeiro, Brazil) for sectioning with a high‐precision metallographic cutter (Isomet 1000; Buehler), equipped with a double‐faced diamond disc (Buehler) operating at 125 rpm with a 100 g load under continuous water cooling. Flat‐parallel root dentine bars measuring 12 mm × 2 mm × 1 mm were obtained, with their dimensions carefully verified using a digital calliper (Carl Mahr Esslingen GmbH, Göttingen, Germany). The bars were stored in plastic containers with distilled water and maintained in an incubator at 37°C under controlled humidity until testing.

The dentine bars from each group were subjected to a three‐point flexural strength test. Each bar was positioned on a support in a Universal Testing Machine (Instron Model 4444; Norwood, MS, USA) with two support points spaced 10 mm apart. A third force application point, 1 mm in diameter, was placed at the midpoint between the supports, applying a constant speed of 0.5 mm/min until the specimen fractured [21, 22]. The maximum force at fracture was recorded. Flexural strength was calculated using the formula: δf = 3PL^2^ / 2bh^2^, where *P* represents the load (*N*) at the peak of the load‐deflection curve, L is the span between support points (10.0 mm), b is the width (2.00 mm), and h is the thickness (1.00 mm) of the dentine bar.

#### 2.3.2 Vickers Microhardness

For the Vickers microhardness test, five roots were selected and longitudinally sectioned using a high‐precision metallographic cutter (Isomet 1000; Buehler), yielding two hemisections per root, for a total of 10 specimens. As this is a non‐destructive test, assessments were performed on the same specimens at three distinct time points (*n* = 10): (T0) non‐irradiated; (T1) after a total radiation dose of 30 Gy; and (T2) after a total radiation dose of 50 Gy. The test was conducted both before and after irradiation, allowing each specimen to serve as its own control.

Longitudinal sections were embedded in epoxy resin and polished. A Vickers microhardness tester (HMV Shimadzu) applied a 100 g load for 15 s on each root third. Pre‐ and post‐irradiation values were recorded. The Vickers microhardness value was calculated using the formula: Vickers = *P*/*A*, where *P* represents the maximum load and *A* is the sensitivity of the depth detection instrument of the equipment [4].

### 2.4 Morphological, Microstructural and Chemical Analyses of Root Dentine

#### 2.4.1 Scanning Electron Microscopy (SEM)

Ten roots were selected and analysed under scanning electron microscopy (SEM) (JEOL JSM 6390 LV, Akishima, Japan). The roots were longitudinally sectioned into two hemisections using a high‐precision metallographic cutter (Isomet 1000; Buehler). Five hemisections were assigned to the RT30Gy group and five to the RT50Gy group. Each hemisection was subjected to the corresponding radiation regimen for its respective group. The homologous halves of the irradiated hemisections served as the control group (NRT—non‐irradiated).

The specimens were immersed in a 1% sodium hypochlorite solution (Asfer, Santa Maria, RS, Brazil) for 5 min, followed by an ultrasonic bath (Ultrasonic Cleaner 1440D; Odontobrás) in distilled water for an additional 5 min. They were then fixed in a 2.5% glutaraldehyde solution buffered with 0.2 M sodium cacodylate for 24 h at 4°C. Next, the specimens were immersed in a 0.2 M sodium cacodylate solution for 1 h, with the solution being replaced every 20 min, followed by a brief rinse with deionised water.

Subsequently, the specimens underwent sequential dehydration in ethanol baths (30%, 50%, 70%, 90%, 95%) for 5 min each, followed by three final baths of 10 min each in 100% ethanol. Finally, the specimens were placed in an oven for 48 h to ensure complete drying. They were then mounted on aluminium stubs and coated with a 300 Å layer of gold–palladium alloy in a vacuum chamber.

The images of the root dentine surface were obtained using SEM under high vacuum (10–15 kV) by a single trained, calibrated, and blinded operator. Nine micrographs were captured per specimen—three from each radicular third (cervical, middle, and apical)—at magnifications of ×500, ×1000, and ×3000. Morphological alterations were qualitatively analysed using a predefined scoring system.

The images were evaluated by two previously calibrated and blinded examiners. The findings observed at different time points were subjected to the Kappa test to assess intra‐ and inter‐examiner agreement, ensuring indices > 0.75 (excellent) for validation and reproducibility. To minimise bias, SEM image analysis was conducted at separate time points, with a 15‐day interval between assessments.

For the dentinal tubules, the following events were considered: 0—intact and regular dentine morphology; 1—dentine morphology with partially obliterated tubules; and 2—dentine morphology with totally obliterated tubules. The presence of cracks and/or fissures in the substrate was classified as: 0—absent and 1—present [10].

#### 2.4.2 Raman Spectroscopy

Five roots were used to assess the chemical composition of root dentine before and after radiotherapy using Raman spectroscopy (Cora 5200, Anton Paar, São Paulo, SP, Brazil). Each root was longitudinally sectioned into two halves (*n* = 10) using a high‐precision metallographic cutter (Isomet 1000; Buehler). As a non‐destructive analysis, the assessment was performed longitudinally at three distinct time points: (T0) non‐irradiated, (T1) after a total radiation dose of 30 Gy, and (T2) after a total radiation dose of 50 Gy.

Between each analysis time point, the specimens were polished (Politriz DP‐10; Panambra) with #1200‐grit sandpaper under constant water cooling. After polishing, they were washed in an ultrasonic bath (Ultrasonic Cleaner 1440D; Odontobrás) with distilled water for 5 min.

The spectra were collected in the spectral range between 400 and 1800 cm^−1^ with a resolution of 4 cm^−1^ and an excitation wavelength of 785 nm. The laser was operated at a power of 21 mW, and the exposure time was 5 s, generating the spectrum. The specimens were positioned with the surface to be analysed facing down (lumen of the canal). Three measurements were taken in the intracanal region of each specimen. After obtaining the spectra, the baseline was corrected and normalised, and the peak areas were identified using OriginPro 2018 software (OriginLab Corporation, Northampton, MA, USA). For analysis of the inorganic composition, the vibrational modes of carbonate ($\nu_{3}{\mathrm{CO}}_{3}^{2-}$) and phosphate ($\nu_{1}{\mathrm{PO}}_{4}^{3-}$; $\nu_{2}{\mathrm{PO}}_{4}^{3-}$; $\nu_{4}{\mathrm{PO}}_{4}^{3-}$) were used, and for the organic composition, the vibrational modes of amide I, CH_2_, and amide III. After obtaining the data for the specific bands corresponding to the inorganic and organic content of the root dentine, the mean value for each specimen was calculated, followed by the group mean (T0, T1, T2) for statistical analysis.

### 2.5 Statistical Analysis

Statistical analysis was conducted using SPSS Statistics version 25.0 software (IBM Corp., Armonk, NY, USA). Data normality was confirmed using the Shapiro–Wilk test, and homogeneity was verified with Levene's test. The effects of radiotherapy and root thirds on Vickers microhardness values were assessed using two‐way ANOVA followed by the Bonferroni post hoc test. The effect of radiotherapy on flexural strength was evaluated using one‐way ANOVA with Tukey's post hoc test. For the Raman data, intergroup comparisons (non‐irradiated, 30 and 50 Gy) were performed using one‐way ANOVA. A significance level of 5% was set for all analyses. Morphological and microstructural changes observed in SEM were qualitatively evaluated.

## 3 Results

### 3.1 Flexural Strength

Table 1 presents the mean and standard deviation values of the flexural strength test. Flexural strength was not affected by radiotherapy, as no statistically significant differences were observed between the experimental and control groups (*p* = 0.998).

**TABLE 1:** Flexural strength mean (MPa) and standard deviation values according to different radiotherapy regimens.

Group	Flexural strength
NRT	208.8 ± 49.1^A^
RT30Gy	210.4 ± 46.4^A^
RT50Gy	210.3 ± 67.9^A^
*p*	0.998

*Note:* Different uppercase letters indicate a statistically significant difference (one‐way ANOVA and Tukey's post hoc test, *α* = 0.05).

Abbreviations: NRT, negative control (non‐irradiated); RT30Gy, total radiation dose delivered to the upper molars simulating radiotherapy for oropharyngeal cancer; RT50Gy, total radiation dose delivered to the upper molars simulating radiotherapy for maxillary cancer.

### 3.2 Vickers Microhardness

Table 2 presents the mean and standard deviation values of the Vickers microhardness. Microhardness was not affected by the different radiotherapy regimens. No statistically significant differences were observed among the groups in the cervical (*p* = 0.092), middle (*p* = 0.616), and apical thirds (*p* = 0.229). Additionally, the root thirds did not affect the microhardness values in any of the experimental conditions: NRT (*p* = 0.183), RT30Gy (*p* = 0.317), and RT50Gy (*p* = 0.549).

**TABLE 2:** Vickers microhardness mean and standard deviation values according to different radiotherapy regimens and root thirds.

	NRT	RT30Gy	RT50Gy	*p*
Cervical	30.0 ± 4.4^A,a^	34.4 ± 6.2^A,a^	36.7 ± 8.0^A,a^	0.092
Middle	35.7 ± 6.4^A,a^	38.6 ± 6.6^A,a^	37.8 ± 5.6^A,a^	0.616
Apical	33.0 ± 7.8^A,a^	38.2 ± 5.3^A,a^	34.5 ± 9.2^A,a^	0.229
*p*	0.183	0.317	0.549	

*Note:* Different uppercase letters in the rows indicate a statistically significant difference with respect to the radiotherapy protocol. Different lowercase letters in the columns indicate a statistically significant difference with respect to the root thirds (two‐way ANOVA and Bonferroni post hoc test, *α* = 0.05).

Abbreviations: NRT, negative control (non‐irradiated); RT30Gy, total radiation dose delivered to the upper molars simulating radiotherapy for oropharyngeal cancer; RT50Gy, total radiation dose delivered to the upper molars simulating radiotherapy for maxillary cancer.

### 3.3 Morphological and Microstructural Assessment (SEM)

**FIGURE 4:**
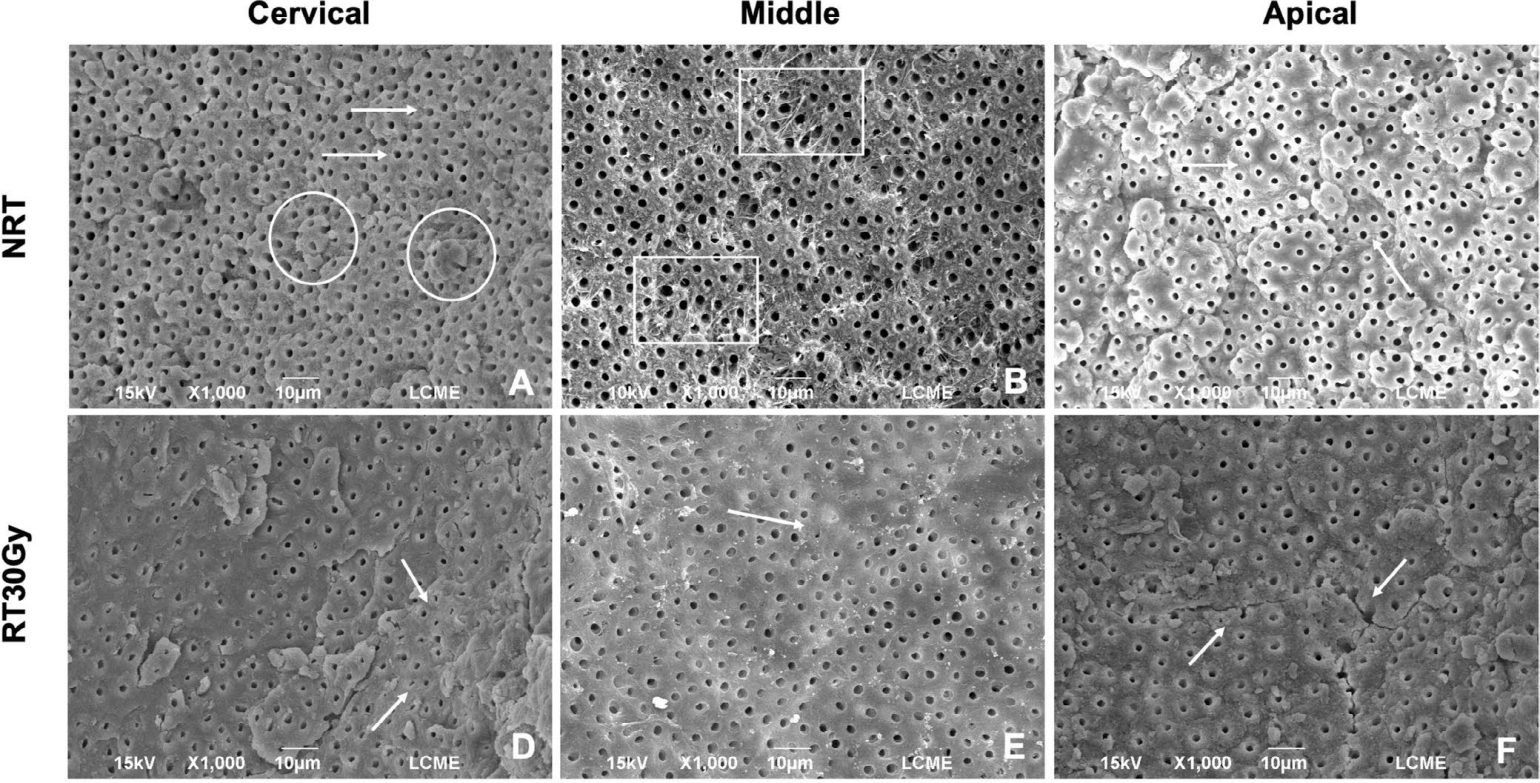
Group NRT: (A) Open dentinal tubules with well‐defined contours (arrows). Presence of calcospherites (mineralization globules) in the non‐instrumented root dentine (circles). (B, C) Intact peri‐ and intertubular dentine, without signs of fissures or cracks, and open dentinal tubules (arrows). Presence of intact collagen fibrils (box) (×1000). Group RT30Gy: (D, E) Partially obliterated dentinal tubules (arrows). (F) Presence of fissures and signs of dehydration (arrows) (×1000).

Intra‐ and inter‐examiner agreement was considered excellent (0.85 and 0.80, respectively). Figure 4 presents representative images of specimens from the RT30Gy group and its corresponding control group (NRT). Differences in the morphology and microstructure of root dentine were observed before and after radiation in all root thirds. The NRT group exhibited more intact and regular root dentine (score 0), with open dentinal tubules and well‐defined contours (score 0) and intact collagen fibrils. In contrast, the RT30Gy group displayed morphological and microstructural alterations compared to the NRT group, including some partially obliterated tubules (score 1) and the presence of fissures in the intertubular dentine (score 1).

**FIGURE 5:**
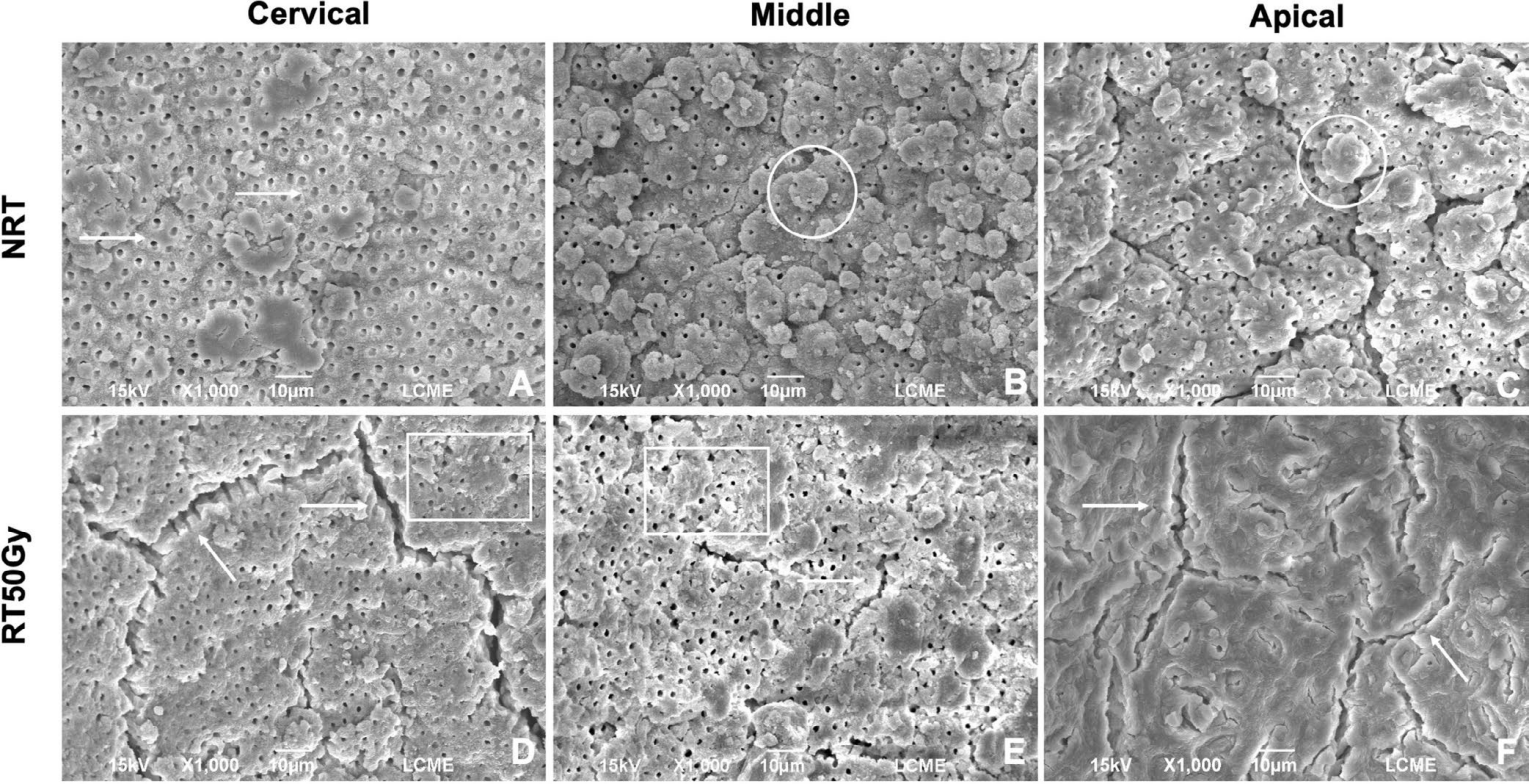
Group NRT: (A) Open dentinal tubules with well‐defined contours (arrows). (B, C) Presence of calcospherites (circles). Intact peri‐ and intertubular dentine without signs of fissures or cracks (×1000). Group RT50Gy: (D, E) Partially obliterated dentinal tubules (box). Intense area of dentine substrate dehydration, with more pronounced fissures and cracks (arrows). (F) Fully obliterated dentinal tubules. Fissures and cracks (arrows) (×1000).

Figure 5 presents representative images of specimens from the RT50Gy group and its corresponding control group (NRT). The morphological and microstructural differences in root dentine before and after radiation were more pronounced. The NRT group exhibited intact and regular morphology (score 0), with well‐defined dentinal tubules (score 0). In contrast, the RT50Gy group showed a more dehydrated dentine substrate, with more pronounced fissures and cracks (score 1) and fully obliterated tubules in most specimens (score 2).

### 3.4 Chemical Analysis (Raman Spectroscopy)

**FIGURE 6:**
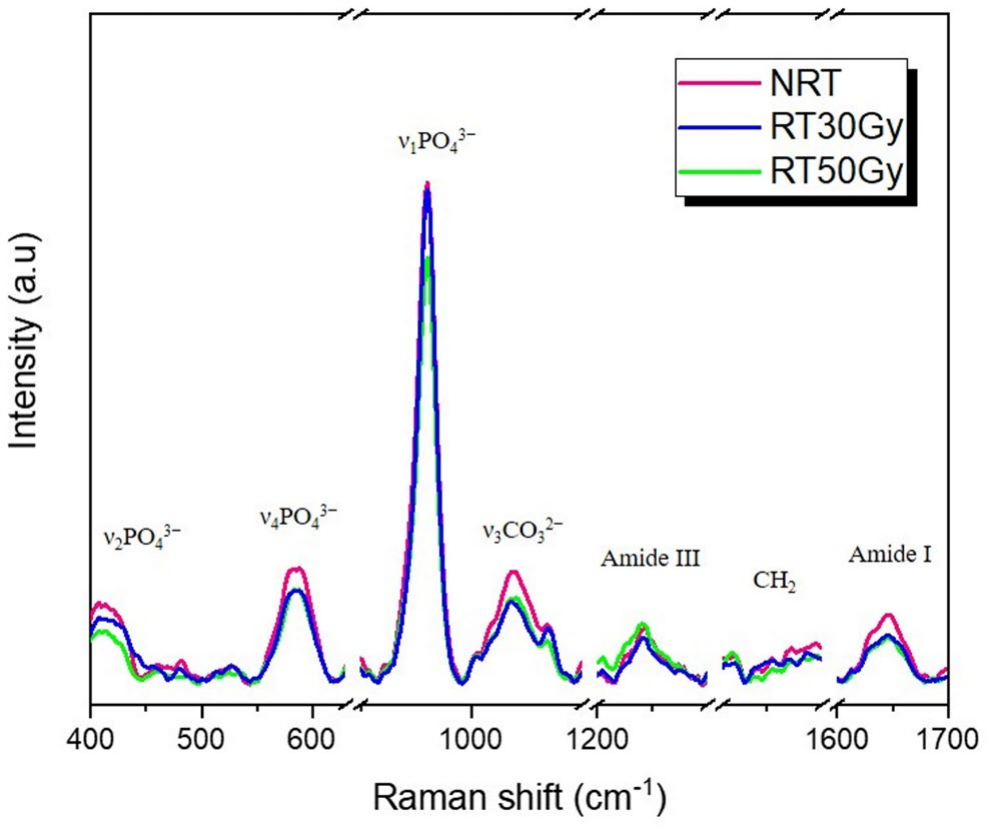
Raman spectra collected from non‐irradiated root dentine specimens (NRT), irradiated with 30 Gy (RT30Gy), and 50 Gy (RT50Gy). Inorganic peaks: $\nu_{2}{\mathrm{PO}}_{4}^{3-}$ (430 cm^−1^), $\nu_{4}{\mathrm{PO}}_{4}^{3-}$ (590 cm^−1^), $\nu_{1}{\mathrm{PO}}_{4}^{3-}$ (960 cm^−1^), and $\nu_{₃}{\mathrm{CO}}_{3}^{2-}$ (1070 cm^−1^). Organic peaks: Amide III (1265 cm^−1^), CH_2_ (1450 cm^−1^), and amide I (1667 cm^−1^).

The intensity of the peaks for the inorganic compounds was centered at 1070 cm^−1^ ($\nu_{₃}{\mathrm{CO}}_{3}^{2-}$ $\nu_{₃}{\mathrm{CO}}_{3}^{2-}$), 430 cm^−1^ ($\nu_{2}{\mathrm{PO}}_{4}^{3-}$), 590 cm^−1^ ($\nu_{4}{\mathrm{PO}}_{4}^{3-}$), and 960 cm^−1^ ($\nu_{1}{\mathrm{PO}}_{4}^{3-}$). The peaks at 960 cm^−1^, 430 cm^−1^, and 590 cm^−1^ are attributed to the ν_1_, ν_2_, and ν_4_ vibrational modes of the phosphate group, respectively. The peak at 1070 cm^−1^ is attributed to the ν_1_ vibration of the carbonate group (carbonate type B), while the peaks for the organic compounds were observed at 1265 cm^−1^ (amide III), 1450 cm^−1^ (CH_2_), and 1667 cm^−1^ (amide I). The ratio between these intensity peaks was calculated for each mapping point of the Raman spectra collected (Figure 6).

Table 3 presents the statistical values for the compounds analysed before and after the different radiotherapy regimens. No statistically significant differences were observed regarding the organic and inorganic components of the root dentine before and after radiation, regardless of the radiotherapy regimen (*p* > 0.001).

**TABLE 3:** Comparison of the integrated area of the Raman bands related to the organic and inorganic content among experimental and control groups.

Comparison among groups	Peak	*p*
NRT vs. RT30Gy vs. RT50Gy	$\nu_{1}{\mathrm{PO}}_{4}^{3-}$ phosphate	0.8307
NRT vs. RT30Gy vs. RT50Gy	$\nu_{2}{\mathrm{PO}}_{4}^{3-}$ phosphate	0.2297
NRT vs. RT30Gy vs. RT50Gy	$\nu_{4}{\mathrm{PO}}_{4}^{3-}$ phosphate	0.3016
NRT vs. RT30Gy vs. RT50Gy	Carbonate	0.1505
NRT vs. RT30Gy vs. RT50Gy	Amide I	0.2374
NRT vs. RT30Gy vs. RT50Gy	CH_2_	0.0835
NRT vs. RT30Gy vs. RT50Gy	Amide III	0.1048

*Note:* No significant differences were observed in the inorganic and organic peaks before and after radiation.

Abbreviations: NRT, negative control (non‐irradiated); RT30Gy, total radiation dose delivered to the upper molars simulating radiotherapy for oropharyngeal cancer; RT50Gy, total radiation dose delivered to the upper molars simulating radiotherapy for maxillary cancer.

## 4 Discussion

This in vitro study assessed the effects of clinically realistic ionising radiation doses, typically received by upper molars during maxillary and oropharyngeal cancer treatment, on root dentine. Based on the results, the null hypotheses were either accepted or rejected according to the specific outcome variable tested. The null hypotheses related to the chemical composition and mechanical properties of root dentine were accepted, as no statistically significant differences were detected among the groups. In contrast, the null hypothesis related to morphology was rejected, since SEM analysis demonstrated dose‐dependent microstructural alterations in irradiated root dentine.

In vitro studies should closely replicate clinical scenarios to avoid overestimating the effects of radiotherapy on dental structures [24]. Understanding these alterations is essential for optimising treatment strategies and improving outcomes in cancer patients [24]. Although the present model simulated clinically relevant radiotherapy doses, interpretation of the findings must consider that dental structures do not necessarily receive the full tumour‐prescribed dose. For instance, in nasopharyngeal cancer irradiated with 60 Gy, lower premolars may receive approximately 30 Gy [18], whereas in oropharyngeal cancers, exposure to the same region may approach 63 Gy [18]. These clinical dose variations are crucial for understanding the biological relevance of the changes observed in vitro.

Radiation‐induced alterations in dentine are dose‐dependent and multifactorial. Lieshout and Boots [25] demonstrated minimal structural damage at ≤ 30 Gy, a two‐ to threefold increase at 30–60 Gy, and nearly tenfold greater alterations above 60 Gy. Moreover, the effects of radiotherapy extend beyond direct tissue damage; treatment‐related changes such as hyposalivation and increased susceptibility to radiation‐related caries play important roles in the clinical deterioration of dentine and the predisposition to pulp and periapical disease [26]. These dynamic and interrelated processes cannot be fully reproduced in vitro and should be considered when interpreting the present findings.

To simulate the oral environment, irradiated specimens are often immersed in artificial saliva, distilled water, or buffered solutions [4, 13, 27, 28]. In the present study, specimens were immersed in distilled water during irradiation to favor radiolysis with minimal chemical interference [29], maintaining hydration and ensuring accurate dose delivery without exogenous ions [5, 10]. After irradiation, the specimens were transferred to artificial saliva to mimic the oral cavity and promote mineral replenishment [29]. Furthermore, in the present study, radiation doses were estimated from dosimetric maps [18], considering tumor site and expected exposure, as prior studies confirm that dental doses vary with cancer location and staging [15, 18, 19]. Applying maximum therapeutic doses (60–70 Gy) in vitro risks overestimation [24] and may obscure true dose–response relationships [24], underscoring the importance of using clinically relevant dosimetry.

Mechanical behaviour was assessed through flexural strength and microhardness tests, which are key indicators of dentine integrity. The three‐point flexural test evaluates resistance to failure [21, 22], and structural compromise increases brittleness and fracture susceptibility [12, 14]. Some studies reported up to a 32.8% reduction in mechanical strength of teeth post‐radiotherapy [14], attributed to collagen degradation via radiolysis and free radicals' action [14]. However, our findings revealed no significant differences between irradiated and non‐irradiated specimens, consistent with a previous study [12], which also observed no flexural strength changes after 60 Gy. Treatment regimen appears influential, as fractionated lower daily doses (2 Gy/day) have produced more detrimental effects than higher single exposures [29]. This distinction is crucial for reproducibility of clinical conditions: while high single‐dose irradiation may accelerate experimental workflows [29], it does not reflect the temporal dynamics, biological repair limitations, or microenvironmental changes seen in patients undergoing HNC treatment [18, 19]. The use of moderate, clinically realistic cumulative doses in our study (30 Gy and 50 Gy), rather than maximal single exposures, provides a more accurate representation of the mechanical response of irradiated dentine under true therapeutic conditions [24].

Vickers microhardness likewise showed no significant changes, diverging from studies reporting reduced hardness after irradiation [7, 10, 12, 27], which attribute such reductions to dehydration and collagen breakdown [7, 10, 12, 27]. It is important to emphasise that radiation has a more pronounced deleterious effect on highly organic structures, such as dentine [7, 27]. Due to its higher water content, dentine is more vulnerable to the effects of radiation [27]. Interestingly, a study [4] observed increased hardness after 72 Gy, likely related to dentine anisotropy and mineral variability [28]. Despite dentine's susceptibility due to high water content [27], in the present study, neither 30 Gy nor 50 Gy radiotherapy regimens had a negative impact on the mechanical properties of root dentine.

Although mechanical and chemical properties were maintained, SEM revealed dose‐dependent morphological degradation. At 30 Gy, cracks were observed, while at 50 Gy, fissures and tubule obliteration became more extensive. These alterations likely reflect collagen network disruption [4, 27] and have been described previously [10], reinforcing that even clinically relevant lower doses [18] can damage dentine microstructure. Raman spectroscopy corroborated the mechanical findings, as no significant modifications were observed in phosphate, carbonate, amide I, CH_2_, or amide III peaks, consistent with an earlier study [8]. Significant reductions in carbonate and amide III were noted only after 60 Gy [8], suggesting that the preservation of organic and inorganic components at 30–50 Gy may explain the mechanical stability observed [4].

As any in vitro investigation, this study has inherent limitations, especially the inability to reproduce oral microbiota, salivary dynamics, and dietary factors, which restricts the extrapolation of our findings to clinical conditions. The mechanical properties of root dentine remained stable in this in vitro model; however, the medium‐ and long‐term clinical behaviour of irradiated dentine is strongly influenced by the altered oral environment that follows radiotherapy [26]. Hyposalivation, microbiota dysbiosis, reduced buffering capacity, and increased cariogenic challenge accelerate dentine demineralization and collagen breakdown, promoting radiation‐related caries and weakening the dentine substrate over time [26]. These post‐radiotherapy conditions also enhance susceptibility to microcrack propagation and structural collapse under functional loading [10, 12]. As a result, even if bulk mechanical properties appear preserved shortly after irradiation, progressive deterioration in vivo may compromise bonding performance and the long‐term stability of endodontic and restorative treatments [23, 30]. This divergence between short‐term in vitro stability and clinical long‐term degradation underscores the importance of considering the dynamic oral environment when interpreting mechanical outcomes of irradiated dentine.

In addition, the literature remains inconsistent regarding radiotherapy's effects on dental tissues [25], largely due to variability in dose distribution and methodological protocols. Future investigations should employ dosimetry that reflects real clinical exposures [15, 18, 19], as standardised, clinically relevant protocols will better simulate patient conditions and improve translational value [24]. This study is the first to simulate average doses typically received by upper molars during maxillary and oropharyngeal cancer treatment, demonstrating that 30 and 50 Gy did not significantly affect dentine's mechanical or chemical properties, although SEM revealed dose‐dependent morphological changes.

Ultimately, although bulk mechanical and chemical integrity was preserved, the microstructural alterations identified may impair adhesion and compromise root canal sealing, posing potential risks to restorative success in cancer patients. Clinicians should recognise this subtle yet clinically significant damage when planning endodontic and restorative procedures in irradiated dentine.

## Author Contributions

All the authors contributed to the study conceptualization and design. **Anna Victória Costa Serique:** conceptualization, investigation, data curation, formal analysis, writing – original draft. **Lívia Ribeiro:** investigation, data curation, formal analysis, writing – review and editing. **Julia Menezes Savaris:** investigation, data curation, formal analysis, writing – review and editing. **Luíz Carlos de Lima Dias‐Júnior:** data curation, formal analysis, writing – review and editing. **Eduardo Antunes Bortoluzzi:** investigation, writing – review and editing. **Mariana Comparotto Minamisako:** formal analysis, writing – review and editing. **Paulo Marcelo Rodrigues:** formal analysis, writing – review and editing. **Nayara Cardoso Cábia:** formal analysis, writing – review and editing. **Ricardo Machado:** investigation, writing – review and editing. **Bruno Alexandre Pacheco de Castro Henriques:** writing – review and editing. **Cleonice da Silveira Teixeira:** investigation, writing – review and editing. **Lucas da Fonseca Roberti Garcia:** conceptualization, investigation, data curation, formal analysis, project administration, writing – original draft, writing – review and editing.

## Funding

This work was supported by Coordenação de Aperfeiçoamento de Pessoal de Nível Superior, 001; Conselho Nacional de Desenvolvimento Científico e Tecnológico, 404289/2023‐1, 306163/2024‐1.

## Ethics Statement

This in vitro experimental study was previously approved by the Ethics Committee for Human Research at the Federal University of Santa Catarina (CAAE: 66249622.1.0000.0121). The study design was conducted following the ethical standards laid down in the 2008 Declaration of Helsinki.

## Conflicts of Interest

The authors declare no conflicts of interest.

## Data Availability

Data is available on request from the authors.
